# Noninvasive Intracranial Pressure Monitoring: Are We There Yet?

**DOI:** 10.1007/s12028-024-01951-1

**Published:** 2024-03-01

**Authors:** Venkatakrishna Rajajee

**Affiliations:** Departments of Neurosurgery and Neurology, University of Michigan, 3552 Taubman Health Care Center, SPC 5338, 1500 E. Medial Center Drive, Ann Arbor, MI 48109-5338 USA

**Keywords:** Intracranial pressure, Transcranial Doppler ultrasonography, Optic nerve, Pupillary reflex, Acute brain injuries

## Abstract

There is an urgent unmet need for a reliable noninvasive tool to detect elevations in intracranial pressure (ICP) above guideline-recommended thresholds for treatment. Gold standard invasive ICP monitoring is unavailable in many settings, including resource-limited environments, and in situations such as liver failure in which coagulopathy increases the risk of invasive monitoring. Although a large number of noninvasive techniques have been evaluated, this article reviews the potential clinical role, if any, of the techniques that have undergone the most extensive evaluation and are already in clinical use. Elevations in ICP transmitted through the subarachnoid space result in distension of the optic nerve sheath. The optic nerve sheath diameter (ONSD) can be measured with ultrasound, and an ONSD threshold can be used to detect elevated ICP. Although many studies suggest this technique accurately detects elevated ICP, there is concern for risk of bias and variations in ONSD thresholds across studies that preclude routine use of this technique in clinical practice. Multiple transcranial Doppler techniques have been used to assess ICP, but the best studied are the pulsatility index and the Czosnyka method to estimate cerebral perfusion pressure and ICP. Although there is inconsistency in the literature, recent prospective studies, including an international multicenter study, suggest the estimated ICP technique has a high negative predictive value (> 95%) but a poor positive predictive value (≤ 30%). Quantitative pupillometry is a sensitive and objective method to assess pupillary size and reactivity. Proprietary indices have been developed to quantify the pupillary light response. Limited data suggest these quantitative measurements may be useful for the early detection of ICP elevation. No current noninvasive technology can replace invasive ICP monitoring. Where ICP monitoring is unavailable, multimodal noninvasive assessment may be useful. Further innovation and research are required to develop a reliable, continuous technique of noninvasive ICP assessment.

## Introduction

- A 37-year-old woman is comatose following an acetaminophen overdose. Her Glasgow Coma Scale (GCS) score is 4T. Computed tomography (CT) of the brain reveals diffuse sulcal effacement. There is concern for cerebral edema and elevated intracranial pressure (ICP), but her international normalized ratio is 3.2, and invasive ICP monitor placement is considered too risky.
- A 22-year-old man is admitted to the intensive care unit in a lower middle-income country with severe traumatic brain injury (TBI). His GCS score is 6T. CT of the brain reveals extensive bifrontal contusions and diffuse sulcal effacement. Neither the hospital nor any other centers in the region have the resources to perform invasive ICP monitoring.

These are two examples that highlight a major unmet need in neurocritical care (NCC). These patients may achieve a good long-term recovery if they survive, without secondary injury from cerebral herniation and compromised brain perfusion. Invasive monitoring of ICP and cerebral perfusion pressure (CPP) is integral to the management of conditions such as severe TBI [1] but is unavailable in many settings, including many low- and middle-income nations. Even when available, conditions such as coagulopathy may present an unacceptably high risk in patients otherwise likely to benefit from invasive monitoring, as in the setting of acute liver failure (ALF) [2]. Findings such as an abrupt drop in the GCS score, absent pupillary light response (PLR), or effacement of the basal cisterns on CT may indicate impending or ongoing cerebral herniation. However, the clinical examination and imaging are insufficiently sensitive and specific to detect ICP elevation above recommended thresholds for treatment [3].

The potential for noninvasive technology extends beyond settings where invasive monitoring is unavailable. Invasive monitoring is associated with risk (bleeding and infection) and cost and typically requires neurosurgical expertise. Noninvasive technology that can replace invasive monitoring will have access to a market valued at more than $1.5 billion [4]. In this context, the absence of reliable noninvasive ICP assessment technology is striking. Although the skull is an obvious barrier to direct noninvasive pressure measurement, decades of research have failed to develop a reliable indirect measure of ICP.

The longstanding quest for a definitive noninvasive ICP monitoring substitute, sometimes considered the “holy grail” of NCC, has generated a large number of potential candidates [5]. The purpose of this article is not to review all these techniques, which are mostly investigational, but to evaluate the clinical role, if any, of the three best-evaluated contenders, which have already been incorporated into clinical practice in many settings: optic nerve sheath diameter (ONSD) measurement, transcranial Doppler (TCD), and quantitative pupillometry.

### Evaluation of Noninvasive ICP Monitoring Technology

Clarity on the proposed clinical role of any new noninvasive device is essential [6]. The requirements of a device used for triage are distinct from the requirements of a device that replaces standard-of-care invasive monitoring. The former requires excellent sensitivity and good specificity, whereas the latter requires excellent sensitivity and specificity relative to the gold standard. Sensitivity may also be most important, with some compromise on specificity acceptable, when the gold standard is unavailable, given the life-threatening consequences of ICP elevation.

Studies of the diagnostic accuracy of noninvasive ICP monitoring techniques are especially susceptible to some of the potential sources of bias listed in the Quality Assessment of Diagnostic Accuracy Studies (QUADAS-2) checklist [7]. The noninvasive (index) test should be compared to the gold standard (invasive monitoring) and not an imperfect surrogate, such as brain imaging or lumbar cerebrospinal fluid pressure. The index test should ideally be performed simultaneously with gold standard invasive measurement. The individual performing the noninvasive measurement should be blinded to the gold standard, particularly when the results of the noninvasive test may be operator dependent. For quantitative tests such as ONSD measurement, following initial exploratory analysis, a consistent threshold should be used and validated across studies. Following confirmation of diagnostic accuracy, use of the device should ideally be shown to improve patient-centered clinical outcomes. This is most important when a new device will be added on to the standard of care [6]. It is preferred but not essential when the device is used as a screening test to select patients for gold standard invasive monitoring or in settings where the gold standard is unavailable. Of note, invasive monitoring has itself never been demonstrated to improve outcomes in a clinical trial [8] but remains a guideline-recommended intervention on the basis of observational studies that demonstrate a reduction in mortality [1, 9, 10].

It should also be noted that currently available noninvasive techniques cannot reliably provide continuous data and, for that reason alone, cannot replace invasive ICP monitoring. Although serial assessments may mitigate this issue, there is little doubt that ICP can fluctuate greatly, and continuous measurement is the gold standard [11].

### Optic Nerve Ultrasound

**Fig. 1:**
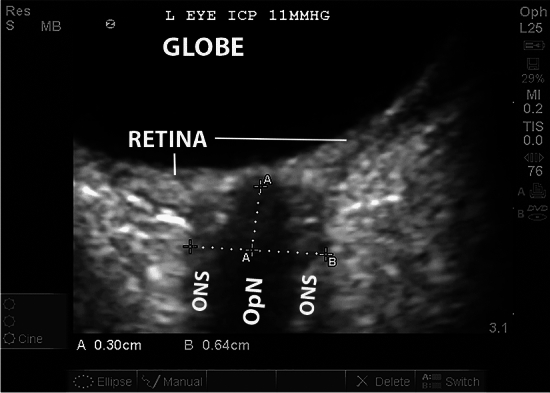
Sonographic measurement of optic nerve sheath diameter (ONSD). Transverse view of the eyeball, with zoomed view of the posterior globe. The globe and retina are visible, with the optic nerve (OpN) visible as a linear densely hypoechoic structure posterior to the globe. The contents of the optic nerve sheath (ONS) are visible on either side of the optic nerve. Caliper A identifies a point 3 mm posterior to the retina, whereas caliper B measures the ONSD at this point at 0.64 cm

The most widely studied noninvasive technique is optic nerve ultrasound, based on the same physiological principles that result in papilledema. The optic nerve sheath (ONS) is a continuation of the dura and contains the subarachnoid space. Elevations in ICP are transmitted through the subarachnoid space and result in distension of the ONS. An ONSD above a predetermined threshold may therefore represent an elevation in ICP [12]. The ONSD can be measured using point-of-care ultrasound machines with a linear transducer and an ophthalmic preset. The eye is commonly imaged in transverse section, and the ONSD measured 3 mm behind the retina (Fig. 1). The optic nerve and the margins of the ONS on either side should be clearly defined. Multiple studies have compared ONSD to concurrent invasive ICP measurement. Although the majority have demonstrated high accuracy [13–19], some, including one study in ALF [20], suggest poor accuracy [21].

**Fig. 2:**
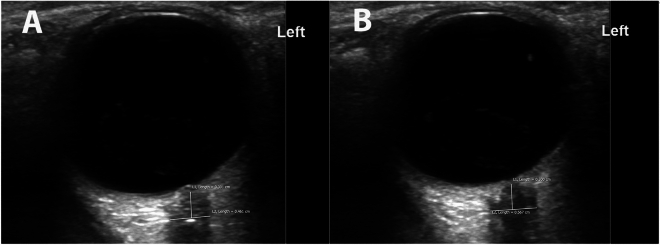
Potential for error in measurement of the optic nerve sheath diameter (ONSD). In panel a, the ONSD is measured at 0.461 cm. However, this is an erroneous measurement from a poor-quality image: the optic nerve and the margins of the optic nerve sheath on either side are not clearly visible. Minimal tilting of the transducer brings these structures into clear focus in panel b, where the ONSD is now measured correctly at 0.567 cm

Several concerns have existed with risk of bias in these studies. Most importantly, few studies have blinded the sonographer to the clinical details of the patient. This may result in errors in definition of the ONS (Fig. 2) based on prior expectations of elevated or normal ICP. The ONSD threshold for diagnosis of elevated ICP has differed across studies. The patient population studied has also varied. The ONS may be impacted differently in TBI compared to chronic hydrocephalus with shunt malfunction. Normal ONSD may vary based on demographics and eyeball size. One preclinical study suggests the ONS may not revert to its original dimensions as quickly following prolonged ICP elevation [22], whereas a clinical study demonstrated lower accuracy when ICP was fluctuating [11].

A recent prospective study addressed some of these sources of bias [23]. Daily ONSD measurement was performed at the point of care (POC) on 120 consecutive patients with severe TBI. In addition, ONSD was measured from deidentified videos by an expert blinded to all clinical details of the patient. Blinding was therefore complete, a standardized measurement protocol was used, and the population was relatively homogenous. In this study, ONSD measurement achieved an area under the curve (AUC) of 0.82 for detection of simultaneous invasive ICP > 22 mm Hg. Serial measurements confirmed a significant correlation. The optimal ONSD threshold was higher, at > 0.72 cm, and previously identified thresholds were inaccurate. Sensitivity was 82%, suggesting one in five genuine ICP elevations may be missed. Although the negative predictive value (NPV) was high at 98%, the positive predictive value (PPV) was poor at 28%. ONSD did not predict the need for higher therapeutic intensity for the treatment of ICP. Most concerning, despite structured training prior to the study, interim analysis demonstrated poor interrater correlation between POC measurements by less experienced sonographers and measurements by blinded experts. Following retraining, high interrater agreement was achieved for the rest of the study.

Although the body of evidence suggests a true biological association between ONSD and ICP, ONSD should likely not serve as the basis for clinical management for several reasons. There is inconsistency in the literature. Erroneous definition of the ONS may occur, particularly with inexperienced sonographers. Most important, a consistent ONSD threshold has not been identified across studies. This threshold may vary by individual, diagnosis, and the degree of ICP fluctuation. Preliminary studies suggest promise with measurement of the globe-to-ONSD ratio [24, 25] and elevation of the optic disk [23], but confirmation in larger studies is necessary.

### Transcranial Doppler

Transcranial Doppler evaluation allows measurement of cerebral blood flow velocities (CBFVs) in large-caliber intracranial arteries and has been used extensively in NCC since Aaslid’s description in 1982 [26]. A wide variety of TCD-based techniques of varying complexity have been developed for the noninvasive evaluation of ICP [27]. Most are primarily investigational, whereas two have been studied more extensively and are used in clinical practice at several centers: the Gosling pulsatility index (PI) [28] and the Czosnyka method of CPP estimation [29]. Both techniques are based on velocity measurements performed primarily in the M1 segments of the bilateral middle cerebral arteries (MCAs). The PI is calculated as follows: (peak systolic velocity—end diastolic velocity)/time-averaged peak velocity. The PI increases with distal resistance to flow, such as with elevated ICP. A low or normal PI may therefore be reassuring. An elevated PI is nonspecific because a variety of factors increase distal resistance, such as chronic small-vessel disease, distal vasospasm, cerebral autoregulatory constriction, and parenchymal pathology, without ICP elevation. Studies of PI for the detection of ICP elevation have had inconsistent results [20, 30–37]. In one recent prospective study of mixed NCC patients, the PI demonstrated an AUC of 0.70 for the detection of invasive ICP > 22 mm Hg [38]. A threshold of 0.82 had high sensitivity (98%), but as expected, specificity was poor at 30%.

In 1998, Marek Czosnyka and colleagues at Cambridge described a technique for estimation of CPP with TCD [29]. Mechanically ventilated patients with moderate/severe TBI underwent continuous TCD evaluation of the bilateral MCAs for 20–120 min with concomitant invasive ICP and intraarterial mean arterial pressure (MAP) monitoring. Time integration of waveform signals for 5-s periods was performed. The estimated CPP was CPPe = [MAP × (CBFV-diastolic/CBFV-mean)] + 14. The estimated ICP was ICPe = MAP − CPPe. In the original study, correlation between CPPe and measured CPP was good (*r* = 0.73; *p* < 10^−6^), with positive predictive power of 94% for detection of CPP < 60 mm Hg. Subsequent studies, however, have demonstrated inconsistent results [20, 33, 39–41]. A study from the same group in 2020 demonstrated disappointing accuracy [39]. More recently, the ICPe technique was evaluated in the Invasive vs. noninvasive Measurement of intracranial PRESSure in brain Injury Trial 2 (IMPRESSIT-2) international multicenter prospective study of diagnostic accuracy [42]. In this study of 262 patients, ICPe demonstrated moderate overall accuracy (AUC 0.76). The NPV for the exclusion of invasive ICP > 22 mm Hg was high (96%), but PPV was low (23%).

It should be noted that most centers use only intermittent TCD evaluation with visual determination of simultaneous ICP and MAP readings, unlike the continuous TCD and digital integration of waveform data used in the original Czosnyka study. In addition, technical considerations impact the accuracy of TCD. In a recent prospective study of 55 mixed NCC patients, the use of transcranial color-coded sonography with correction for angle of insonation improved the AUC of the ICPe method for detection of ICP > 22 mm Hg from 0.51 (sensitivity 71%) to 0.73 (sensitivity 100%), although specificity remained low at 30% [38]. Positioning of the arterial transducer has been at the phlebostatic axis in some studies [29, 38] and at the tragus in others [42], which is expected to result in higher or lower estimates of CPPe, respectively.

**Fig. 3:**
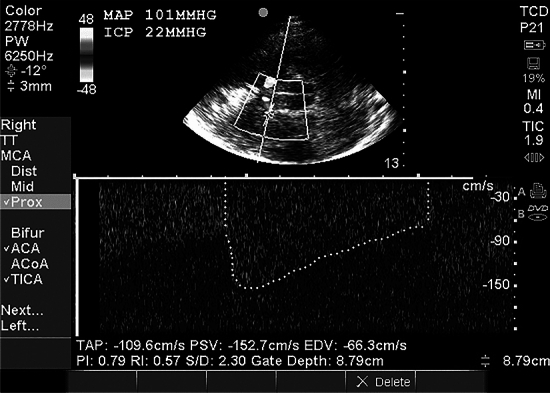
Transcranial color-coded sonography (TCCS) for estimation of intracranial pressure (ICP) in a patient with severe traumatic brain injury. The time-averaged peak velocity (TAP) in the right middle cerebral artery (MCA) is measured at 109.6 cm/s following correction for an angle of insonation of 12°. The end diastolic velocity (EDV) is 66.3 cm/s. Simultaneously measured intraarterial mean arterial pressure (MAP) is 101 mm Hg. Using the Czosnyka formula, the estimated ICP (ICPe) is calculated at 26 mm Hg. The simultaneously measured ICP from the invasive monitor is 22 mm Hg

The ICPe method with angle correction (Fig. 3) and possibly the PI likely have a role in clinical practice to exclude ICP elevation when invasive monitoring is unavailable. ICPe systematically overestimates ICP [38, 43]. An elevated ICPe or PI has a poor PPV and should not, in isolation, trigger therapeutic intervention.

### Quantitative Pupillometry

**Fig. 4:**
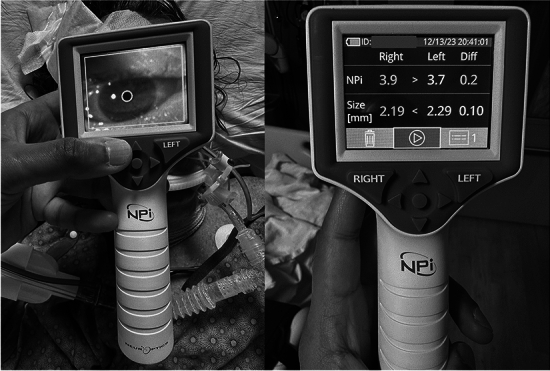
Quantitative pupillometry. The image on the left shows use of the pupillometer, which automatically detects the pupil with image analysis. The image on the right shows measurements of the bilateral neurological pupil index (NPi; NeurOptics, Irvine, CA) and pupillary size with side-to-side difference

A compressive oculomotor nerve palsy has long been considered “nature’s ICP monitor” and a sign of cerebral herniation. A reduction in parasympathetic activity of the nerve as a result of compressive lesions or elevated ICP may result in subtle impairment of pupillary reactivity not easily elucidated by manual testing. Subjective assessment of the PLR is often inaccurate; in one study, up to one third of pupils judged nonreactive by NCC nurses were reactive when assessed with a pupillometer [44]. Quantitative pupillometry uses image analysis software to precisely measure pupillary size at rest using infrared light, speed of constriction following exposure to visible light, final size following maximal constriction, and speed of relaxation. These data are used to generate proprietary indices, such as the neurological pupil index (NPi; NeurOptics, Irvine, CA) [45, 46]. An NPi < 3.0 represents a sluggish pupil, whereas an NPi of 0 denotes nonreactivity. Quantitative pupillometry provides a sensitive and objective assessment of the PLR. Compared to other noninvasive techniques, pupillometry is an intuitive and familiar way for clinicians to assess ICP. An expert sonographer is not required, and frequent assessments can be performed by bedside nursing using a small handheld device (Fig. 4). Uncal herniation that may occur with normal ICP following ischemic stroke or other temporal lobe pathology can be detected at an early stage.

Despite the advantages, there is only a limited body of evidence that confirms the diagnostic accuracy of this tool. Pupillometry demonstrated a significant correlation to invasive ICP measurement in one study [47]. A retrospective study of 65 mixed NCC patients in Sweden demonstrated an AUC of 0.72 with an NPV of 97% for a threshold of NPi < 3.9 to exclude ICP > 20 mm Hg [48].

Quantitative pupillometry is in widespread use globally, is easy to use, and is relatively inexpensive. It is reasonable to integrate this tool into clinical practice for the noninvasive detection of life-threatening ICP elevation.

### Multimodal Assessment and Noninvasive Assessment Protocols

All the noninvasive measures described in this article have been evaluated in relatively small studies, with wide confidence intervals for all measurements of accuracy. Most important, the high NPV in some studies may simply reflect the lower prevalence of ICP elevation [38, 42]. These techniques should, therefore, not be used in isolation; multimodal assessment is required. A 2020 prospective single-center study evaluated all the techniques described in this article [49]. TCD achieved the highest accuracy for detection of invasive ICP > 20 mm Hg. The AUCs were 0.86 for TCD-ICPe, 0.85 for TCD-PI, 0.78 for ONSD, and 0.71 for pupillometry. A combination of these techniques, however, achieved an AUC of 0.91.

We use pupillometry performed every 1–4 h by bedside nursing and daily TCD to monitor patients at high risk for life-threatening ICP elevation but ineligible for invasive monitoring, including patients with ALF. These data are integrated with information from other sources, such as CT imaging and (in patients with ALF) ammonia levels [50]. An NPi > 3.9 [48], along with an average ICPe < 22 mm Hg from the bilateral MCAs [38], is considered reassuring. An ICPe ≥ 22 mm Hg or an NPi of 3.0–3.9 is common but nonspecific; frequent assessment with pupillometry, clinical examination, and intermittent CT imaging is continued. A sluggish (NPi < 3.0) or nonreactive pupil (NPi = 0) should trigger urgent evaluation. A rapid assessment should be performed for possible erroneous measurement or confounders such as mydriatic medications. Urgent CT imaging is often necessary. In the appropriate context, urgent therapeutic measures to reverse herniation should be initiated.

Several newer noninvasive technologies have undergone limited validation. These include novel devices that use sound propagation between the ears [51] or detect pulsatile microdeformations of the skull [52]. While promising, these devices require more extensive validation prior to integration into clinical practice.

## Conclusions

No current noninvasive technology can replace invasive ICP monitoring. Where invasive monitoring is unavailable, however, multimodal assessment with noninvasive techniques may be useful. Further innovation and research are required to develop a reliable, continuous technique of noninvasive ICP assessment.
